# Neutrophils: Old cells in IBD, new actors in interactions with the gut microbiota

**DOI:** 10.1002/ctm2.1739

**Published:** 2024-06-14

**Authors:** Camille Danne

**Affiliations:** ^1^ Sorbonne Université, INSERM UMRS‐938, Centre de Recherche Saint‐Antoine, CRSA, AP‐HP, Hôpital Saint‐Antoine, Service de Gastroentérologie Paris France; ^2^ Paris Center For Microbiome Medicine (PaCeMM) FHU Paris France

**Keywords:** IBD, intestinal inflammation, microbiota, neutrophil

1

Inflammatory bowel disease (IBD) is a chronic inflammatory condition with increasing prevalence. Current therapies are only partially efficient and associated with potentially severe side effects, highlighting the need for innovative therapeutic strategies. Here, we explored the novel dialogue between neutrophils and microbiota in intestinal homeostasis and inflammation, opening a promising avenue for IBD management.^1^

IBD is complex, resulting from genetic predisposition, alterations of the gut microbiota, and environmental factors.^2^ Unlike auto‐inflammatory diseases caused by the spontaneous over‐activation of inflammatory pathways, IBD often involves loss‐of‐function mutations. Deficient innate immune responses, including defects in the intestinal epithelial barrier, fail to control the altered intestinal microbiota, triggering more activation of the adaptive immune system and leading to chronic inflammation and tissue damage.^3^ For example, a loss‐of‐function mutation in the gene NOD2, encoding an innate immunity sensor of bacterial wall components, is associated with Crohn's disease,^4^ whereas a gain‐of‐function mutation in the same gene causes Blau syndrome.

Neutrophil infiltration into the intestinal mucosa is a hallmark of active IBD, with calprotectin and other neutrophil granule proteins as key faecal biomarkers. Recently, the detection of autoantibodies directed against granulocyte–macrophage colony‐stimulating factor (GM‐CSF) was shown to predict complicated ileal Crohn's disease long before the diagnosis.^5^ GM‐CSF is a key regulator of neutrophil production and function, further emphasizing the importance of neutrophils in the development of IBD. However, several limitations have hindered the development of therapeutic strategies targeting these cells. First, they remain challenging to work with. Neutrophils are terminally differentiated cells with a short lifespan, preventing delayed analyses and genetic manipulation. Consequently, their roles and functions in IBD have been poorly investigated compared to other immune cells like T cells. However, recent advances, particularly through high‐throughput technologies, unveil an unexpected complexity, with heterogeneous populations and dual functions, both deleterious and protective for the host.^1^

Progress in comprehension of neutrophil biology in health and disease helps in understanding why targeting neutrophils from a therapeutic perspective might be a double‐edged sword. The risk of severe infections due to neutrophil deficiency raises safety concerns about their pharmacological inhibition or depletion. Additionally, boosting neutrophil activity, while sometimes necessary, could be associated with deleterious consequences of over‐inflammation. This might explain why clinical trials targeting neutrophil‐associated molecules (notably GM‐CSF, matrix metalloproteinase‐9 and the cytokine interleukin [IL]‐17) have not provided the expected results, showing none or limited positive effects.^1^ Additionally, selectively targeting neutrophils is challenging due to their close resemblance to other myeloid cells like monocytes, macrophages, and even osteoclasts. It is essential to develop strategies to increase the selectivity of therapeutic interventions that target neutrophils and aim for modulation instead of inhibition of neutrophil functions.

In this context, interconnections between disease development, intestinal microbiota and neutrophils are highlighted. Indeed, intestinal microbiota alterations are both a cause and a consequence of IBD. IBD is associated with modifications to the composition and functions of the intestinal microbiota,^1^ and the role of the intestinal microbiota as an actor in IBD pathogenesis is now clearly proved. At the intersection between IBD and the intestinal microbiota, neutrophils appear as key players. Numerous IBD susceptibility genes are involved in neutrophil functions related to defence against microbes, including NOD2, NCF4, LRRK2 and CARD9.^1^, ^6^ Moreover, severe monogenic diseases with a functional defect of neutrophils, including Chronic Granulomatous Disease, are characterized by both an intestinal inflammation mimicking IBD and alterations of the intestinal microbiota. It demonstrates the existing dialogue between neutrophils, gut inflammation and microbiota.

From a mechanistic perspective, neutrophils affect the microbiota composition and function by different means^1^, ^7^ (Figure 1). As phagocytic cells, they participate in microbiota containment and limit microbial dissemination during infectious episodes. In the case of prolonged inflammation, massive and chronic neutrophil infiltration modifies the intestinal environment, notably through the release of various molecules, such as reactive oxygen species (ROS) and proteins from granules. This provides a selective advantage to facultative anaerobic bacteria, participating in the expansion of Enterobacteriaceae in the inflammatory context in humans and mice.

**FIGURE 1:**
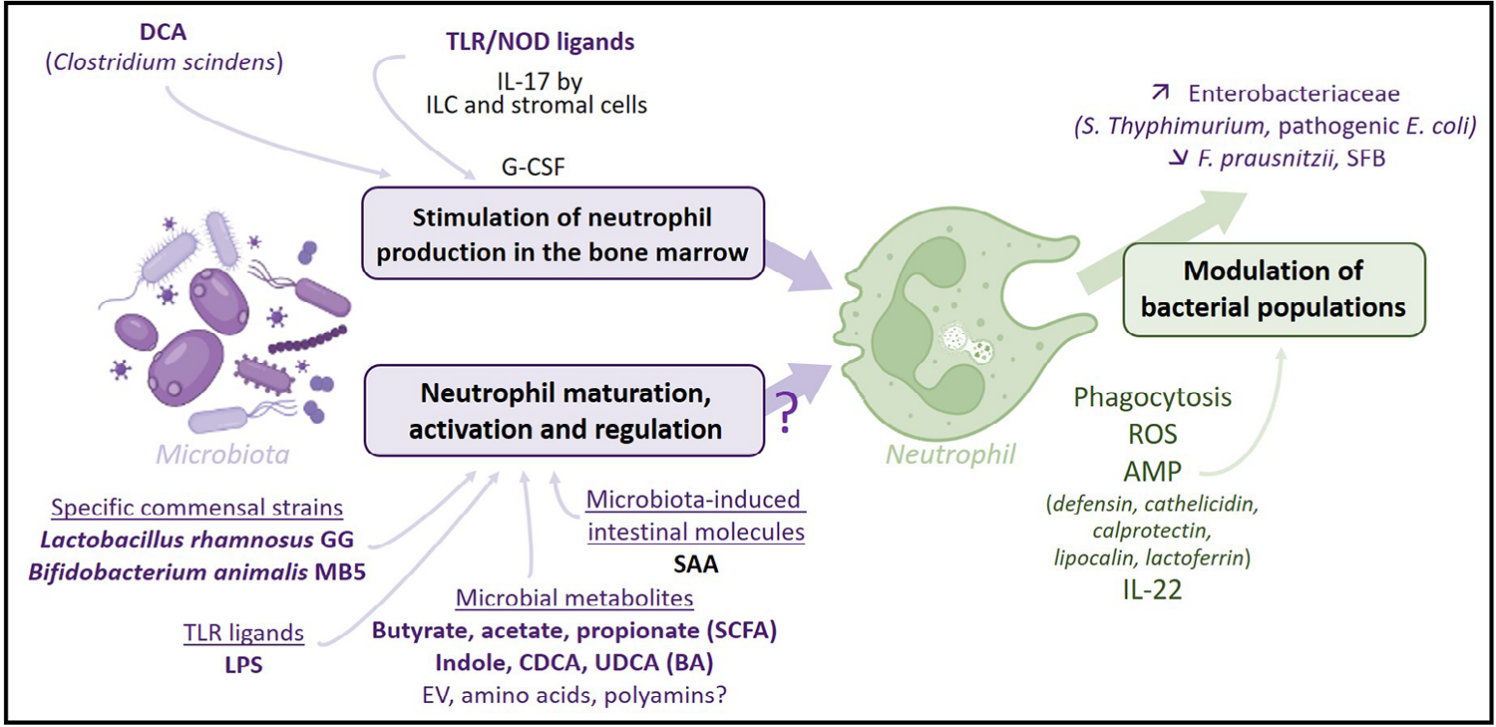
Interactions between neutrophils and the intestinal microbiota. AMP, antimicrobial peptides; BA, bile acids; CDCA, chenodeoxycholic acid; DCA, deoxycholic acid; EV, extracellular vesicles; G‐CSF, granulocyte colony‐stimulating factor; ILC, innate lymphoid cells; LPS, lipopolysaccharide; NOD, NOD‐like receptor; ROS, Reactive oxygen species; SAA, serum amyloid A; SCFA, short‐chain fatty acids; SFB, segmented filamentous bacteria; TLR, Toll‐like receptor; UDCA, ursodeoxycholic acid. Inspired by Danne et al., Neutrophils: from IBD to the gut microbiota, NRGH 2024.

In return, microbiota factors regulate neutrophil production and functions directly and indirectly. The microbiota stimulates neutrophil production in the bone marrow, as evidenced by the significant decrease in neutrophil numbers after antibiotic use and in germ‐free mice.^7^ Administration of killed bacteria or lipopolysaccharide (LPS) in these contexts is sufficient to restore neutrophil production. Indeed, microbial components, but also direct contact between epithelium and certain bacterial species, trigger the secretion of IL‐17 by innate lymphoid cells and stromal cells (Figure 1).^1^, ^7^ IL‐17 induces G‐CSF synthesis, which regulates neutrophil production.^7^ Various microbial ligands, including LPS, also activate neutrophil functions, including phagocytosis, migration and ROS production.^1^, ^7^ Moreover, certain microbial metabolites, such as short‐chain fatty acids^8^, ^9^ and bile acids,^1^, ^7^ were shown to regulate neutrophil activity, promoting their defence functions while reducing their pro‐inflammatory responses. Interestingly, indoles, molecules derived from tryptophan degradation by the microbiota, are selective inhibitors of neutrophil myeloperoxidase activity,^10^ which might partly explain their protective role in both human and murine colitis. However, due to the technical limitations discussed above, findings related to neutrophil‐microbiota dialogue remain scarce and controversial, especially in the IBD context.

These observations suggest that directly targeting the intestinal microbiota to regulate neutrophil functions, in conjunction with other therapies, could be an attractive strategy for managing IBD. Faecal microbiota transfer could restore healthy neutrophil‐microbiota interactions, but challenges, such as reproducibility, scalability, and cost, limit its widespread use in chronic conditions. Dietary intervention could enhance the production of butyrate and other beneficial metabolites by the intestinal microbiota, thereby regulating the activity of neutrophils and the immune cells they interact with. However, clinical trials remain difficult to conduct, especially as dietary intervention involves multiple biases and uncontrolled parameters. In this context, live biotherapeutic products, a novel class of biological products containing live microorganisms and used for the prevention or treatment of human diseases, represent an exciting option.

Going forward, it is crucial to elucidate the fine crosstalk between neutrophils and intestinal microbiota, at steady state and in inflammatory conditions, to develop new ways to tackle complex diseases associated with microbiota alterations, such as IBD.

## AUTHOR CONTRIBUTIONS

Commentary written by Camille Danne based on the publication “Danne et al., Neutrophils: from IBD to the gut microbiota, NRGH 2024”.

## ETHICS STATEMENT

I have no competing interest to declare.
